# Anti-Adalimumab Antibodies Purified from Juvenile Idiopathic Arthritis Patients: Kinetic Characterization Among Biosimilars

**DOI:** 10.3390/bios15050278

**Published:** 2025-04-29

**Authors:** Andrea Di Santo, Edoardo Marrani, Carmen Gallo, Fosca Errante, Valerio Maniscalco, Anna Maria Papini, Gabriele Simonini, Paolo Rovero, Feliciana Real Fernandez

**Affiliations:** 1 Interdepartmental Research Unit of Peptide and Protein Chemistry and Biology (Peptlab), University of Florence, 50019 Sesto Fiorentino, Italy; andrea.disanto@unifi.it (A.D.S.); carmen.gallo@unifi.it (C.G.); fosca.errante@unifi.it (F.E.); annamaria.papini@unifi.it (A.M.P.); 2 Department of Neurosciences, Psychology, Drug Research and Child Health (NeuroFarBa), University of Florence, 50019 Sesto Fiorentino, Italy; gabriele.simonini@unifi.it; 3 Rheumatology Unit, ERN ReCONNET Center, Meyer Children’s Hospital IRCCS, 50139 Firenze, Italy; edoardo.marrani@meyer.it (E.M.); valerio.maniscalco@unifi.it (V.M.); 4 Pediatrics and Neonatology Unit, Santo Stefano Hospital, 59100 Prato, Italy; 5 Department of Chemistry “Ugo Schiff”, University of Florence, 50019 Sesto Fiorentino, Italy; 6 Institute of Chemistry of Organometallic Compounds, National Research Council (ICCOM-CNR), 50019 Sesto Fiorentino, Italy

**Keywords:** adalimumab, surface plasmon resonance, anti-drug antibodies, biosimilars, juvenile idiopathic arthritis

## Abstract

The use of adalimumab biosimilars has become increasingly common in clinical practice, reflecting their growing acceptance and efficacy as therapeutic alternatives to reference biologics. However, studies investigating the molecular interactions between anti-adalimumab antibodies (AAA) elicited in patients and different adalimumab biosimilars remain limited. This study aims to characterize the kinetic interactions between purified AAA from pediatric patients with Juvenile Idiopathic Arthritis and three adalimumab formulations: the originator Humira^®^, and the biosimilars GP2017 (Hyrimoz^®^) and SB5 (Imraldi^®^). For this purpose, adalimumab formulations were immobilized on a gold chip, and purified AAA were flowed to perform further kinetic analysis using the surface plasmon resonance (SPR) technology. Results showed that the K_D_ values for purified AAA from patients treated with biosimilars GP2017 (Hyrimoz^®^) or SB5 (Imraldi^®^) were comparable across all formulations tested, including the originator Humira^®^. AAA interacted with Humira^®^, Hyrimoz^®^, and Imraldi^®^ with similar apparent affinity (10^−9^ M > K_D_ > 10^−10^ M); slight variations have been observed among patients, less among biosimilars. The similarity in K_D_ values across biosimilars and the originator supports the notion that, at the level of immunogenicity, biosimilars can be considered clinically comparable to the originator.

## 1. Introduction

Adalimumab (ADL) is a fully human monoclonal antibody that targets tumor necrosis factor-alpha (TNF-α). By binding to TNF-α, it prevents the cytokine activation cascade produced by the interaction with its receptors on the cell surface, thereby inhibiting the immune and inflammatory responses. The originator, AbbVie’s Humira^®^ drug, approved by the US Food and Drug Administration (FDA) in 2002 and by the European Medicines Agency (EMA) in 2003, was the first commercially available form of adalimumab, used for the therapy of patients with arthritic diseases caused by elevated levels of TNF-α [1]. This drug has been deemed safe and is approved as well for use in pediatric patient pathologies, i.e., Juvenile Idiopathic Arthritis (JIA), Pediatric Crohn’s Disease, and Ulcerative Colitis. The widespread indications and its high cost led to Humira^®^ being the most sold drug worldwide, breaking US$ 20 billion of revenues in 2021 [2]. In 2018, the patent of Humira^®^ expired in the EU and biosimilars entered the market. One year later, 35% of European patients had been switched from adalimumab originator to biosimilars [1,3]. The EMA defined a biosimilar as “… a biological medicine highly similar to another already approved biological medicine (the ‘reference medicine’), approved according to the same standards of pharmaceutical quality, safety and efficacy that apply to all biological medicines” [4]. Similarly, the FDA considers biosimilars “as a biopharmaceutical that is highly similar to an already licensed biologic product notwithstanding minor differences in clinically inactive components and for which there are no clinically meaningful differences in purity, potency, and safety between the two products” [5]. Thus, biosimilar cannot be considered the generic of a biological medicine, mostly because of the natural variability and the more complex manufacturing of biological medicines that do not allow an exact replication of the molecular microheterogeneity, and the development of the two is different. While for generic medicines the bioequivalence is required (i.e., that both the generic and the reference medicine release the active ingredient into the body at the same rate and to the same degree under comparable conditions), in the case of biosimilar, the biosimilarity is required, thorough head-to-head comparison of the biosimilar and the reference medicine to demonstrate strong resemblances in chemical structure, biological function, efficacy, safety, and immunogenicity. Moreover, efficacy and safety must be established for each indication. In fact, the EMA biosimilar approval pathway emphasizes pre-clinical analytic data along with clinical studies to confirm therapeutic equivalence [6]. However, confirmatory clinical trials for the biosimilar are typically not required for every indication approved for the reference medicine: once biosimilarity is demonstrated, data can be extrapolated to other indications if the available scientific evidence sufficiently addresses all relevant aspects of those indications [7]. To date, there are ten biosimilars of Humira^®^ approved in the EU: Amgevita^®^, Amsparity^®^, Hefiya^®^, Hukyndra^®^, Hulio^®^, Hyrimoz^®^, Idacio^®^, Imraldi^®^, Libmyris^®^, and Yuflyma^®^ [8], leading to unprecedented competition among biosimilars themselves and the originator. Reasons for switching from originator adalimumab to an adalimumab biosimilar may rely first of all on providing a lower-cost alternative, but also due to tolerability issues, or as consequences of third parties, such as payers [9]. Thus, the switching from originator to biosimilar should be considered case-by-case depending on a patient’s clinical situation, considering, however, that evidence suggests a reassuring profile of effectiveness when substituting the originator with a biosimilar, despite data about adalimumab still being limited [10,11].

A key parameter in the biological therapy regards the immunogenicity, as anti-drug antibodies have been identified as the major factor contributing to the failure of the treatment [12]. Their hypothesis of action ranges from formation of immunocomplexes, that may enhance the drug clearance, to functional neutralization of the drug, thus interfering with the therapy [13,14,15]. The incidence of anti-drug antibodies during the treatment has been shown to vary depending on different factors such as treatment duration and dosage, but also depending by the detection method used [16,17]. In the case of adalimumab, insurgence of anti-adalimumab antibodies (AAA) have been detected fluctuating from less than 5% to over 80% [18]. Biosimilar should not present clinically meaningful differences in immunogenicity, safety, or effectiveness when compared to originator, thus this propriety is highly investigated during the development of a biosimilar [9]. Despite the similar immunogenicity that a biosimilar should maintain compared to the originator, minimal differences present in the biosimilar may produce slight variations of interactions between the drug and the AAA.

We previously kinetically characterized the interaction between purified AAA from JIA patients treated with the originator Humira^®^ drug using the surface plasmon resonance technique [19]. According to the current state of knowledge, studies on the molecular interactions between AAA and the different forms of adalimumab biosimilars have not been yet performed. In this context, a deep understanding in AAA behavior becomes of critical importance for tracking and understanding how the immune system reacts to biosimilars, which can affect the efficacy and safety of the treatment. In the present study, we aim to characterize kinetic interactions that occur between purified AAA from JIA pediatric patients and three different forms of adalimumab: the originator Humira^®^, and the biosimilars GP2017 (Hyrimoz^®^) and SB5 (Imraldi^®^). For this purpose, the different adalimumab forms will be immobilized on a gold chip to perform a kinetic analysis of purified AAA using the surface plasmon resonance (SPR) technique.

## 2. Materials and Methods

### 2.1. Chemicals and Apparatus

Surface plasmon resonance experiments were performed in a Biacore^®^ X100 instrument from Cytiva^TM^ (Uppsala, Sweden). All solutions were prepared with Milli-Q water obtained using the Sartorius system (Arium^®^ 611 VF) used to prepare solutions, which were filtered daily with a Millipore Express™ PLUS 0.22 μm filtration system. The CM5-type chips, amine coupling kit, 10 mM pH 2.5 glycine solution, and running buffer HBS-EP+ 10× (0.1 M HEPES, 1.5 M NaCl, 30 mM EDTA, 0.5% *v*/*v* surfactant P20) were acquired from Cytiva^TM^ (Uppsala, Sweden). Running buffer was diluted ten times with Milli-Q water at pH 7.4 and filtered daily. Sensor chips with antibodies immobilized on the surface were stored at 4 °C in running buffer. Sodium acetate was purchased from Carlo Erba (Milano, Italy), sodium hydroxide from Honeywell-Riedel deHaen (Seelze, Germany), and sodium chloride from VWR International (Radnor, PE, USA).

The stock solution of Humira^®^ at 100 mg/mL for clinical use was from AbbVie Ltd. (lot n°42296AS09; Maidenhead, UK). Hyrimoz^®^ stock as 50 mg/mL solution for clinical use was from Sandoz GmbH Biochemiestr. (lot n° MK9014; Kundle, Austria). Imraldi^®^ stock 100 mg/mL solution for clinical use was from Samsung Bioepis NL B.V. (lot n°GR423571; Delft, The Netherlands). AffiniPure Donkey Anti-Goat IgG (H+L) was from Jackson ImmunoResearch Europe Ltd. (lot n°YD3901930E; Ely, UK). Cyanogen bromide-activated-Sepharose™ 4B was purchased from Merck (Darmstadt, Germany). Filter membrane Pierce™ Protein Concentrators PES, 30 K MWCO, 2–6 mL were purchased from Thermo Scientific (Waltham, MA, USA). Hydrochloric acid, sodium bicarbonate, and glycine were purchased from Sigma-Aldrich (St. Louis, MO, USA). D-PBS solution was prepared using calcium chloride from Honeywell Fluka (Charlotte, NC, USA), potassium chloride from Merck (Darmstadt, Germany), magnesium chloride and disodium hydrogen phosphate from Carlo Erba (Milan, Italy). Centrifuges were performed on Megafuge 1.0R from Heraeus (Hanau, Germany). Spectrophotometric studies were performed on UV-Vis Cary 4000 from Agilent (Santa Clara, CA, USA).

### 2.2. Sensor Chip Preparation

Experiments were conducted following the previously described SPR protocol properly optimized for the present study [20]. Each CM5-type sensor chip was prepared immobilizing the drug on the flow cell 2 (Fc2) and a reference antibody (Donkey Anti-Goat IgG, Dag-IgG) on the flow cell 1 (Fc1). The antibodies were covalently linked according to the standard amine coupling strategy. Using this protocol, antibodies are randomly oriented on the chip surface. Nevertheless, the detection of binding signals indicates that a sufficient proportion of antibodies retained their functionality. Therefore, we considered the potential signal loss due to a suboptimal orientation to be acceptable for the purposes of this study. The standard pH scouting procedure was employed for the immobilization buffer selection, using the following buffers: sodium acetate buffer 10 mM pH 4.0, 5.0, 5.5, and 6.0; sodium acetate buffer 20 mM pH 5.0 and 5.5; and sodium acetate buffer 50 mM pH 5.0 and 5.5. Adalimumab (ADL) was diluted to a final concentration of 20 μg/mL in each buffer. For the immobilization of Humira^®^, 10 mM sodium acetate at pH 5.0 was selected as the optimal buffer. The sensor chip surface was activated by injecting a 1:1 mixture of 0.1 M N-hydroxysuccinimide (NHS) and 0.4 M 1-ethyl-3-(3-dimethylaminopropyl)-carbodiimide (EDC) for 480 s at a flow rate of 5 μL/min. ADL was then immobilized to a target level of 8000 resonance units (RU). Residual active esters were quenched by injecting 1 M ethanolamine-HCl (pH 8.5) for 60 s at a flow rate of 10 μL/min. The immobilized Humira^®^ raised a final immobilization level of 7600 RU. Hyrimoz^®^ and Imraldi^®^ were immobilized on the Fc2 of a CM5-type sensor chip surface independently according to the protocol described for Humira^®^, using 50 mM sodium acetate pH 5.0 as selected buffer raising a final immobilization level of 7750 and 7863 RU, respectively. AffinityPure Donkey Anti-Goat IgGs were immobilized on the flow cell 1 (Fc1) as a reference according to the same protocol described above, using 50 mM sodium acetate pH 5.0 as selected buffer. The immobilized IgGs raised a final immobilization level of 7750 ± 150 RU.

### 2.3. Patients and Samples

Samples were collected from recruited patients suffering Juvenile Idiopathic Arthritis (JIA) and treated with ADL at the Pediatric Rheumatology Unit of Anna Meyer Children’s University Hospital, Florence, Italy. The study protocol was approved by the Ethical Committee of Meyer Children’s Hospital IRCCS and informed consent was obtained in all cases. The procedures followed in the study were in accordance with the Helsinki Declaration. Blood samples were obtained after centrifugation of sera and stored at −20 °C, and thawed only once before use. Adalimumab and AAA serum levels were measured with the surface plasmon resonance technology following the previously described protocol as part of regular medical care [20]. Two patients presenting high AAA titer were selected among patients in order to purify antibodies for further characterization. Selected patients’ characteristics are summarized in Table 1.

Patient #1, a 17-year-old female, was diagnosed with rheumatoid factor-negative polyarticular juvenile idiopathic arthritis (JIA), with a disease duration of 10 years. She had previously been exposed to adalimumab (originator) for 56 months. At the time of sampling, receiving adalimumab (biosimilar, Imraldi^®^) for 6 months, she was on clinical inactive disease and MTX was not concomitantly administered. At the last available follow-up, after 51 months from starting adalimumab, she was still in clinical disease remission, and no adverse events related to biosimilar were reported. Patient #2, a 10-year-old female, was diagnosed with oligoarticular JIA with a disease duration of 8 years. She had previously been treated with adalimumab (originator) for 22 months. At the time of sampling, she had been receiving adalimumab (biosimilar, Hyrimoz^®^) for 3 months in combination with oral methotrexate (7.5 mg/week) and no sign of disease activity was present. At the last available follow-up, after 50 months from starting adalimumab, she was in disease remission, and no adverse events related to the treatment were reported.

### 2.4. Anti-Drug Antibodies (AAA) Purification

**Figure 1:**
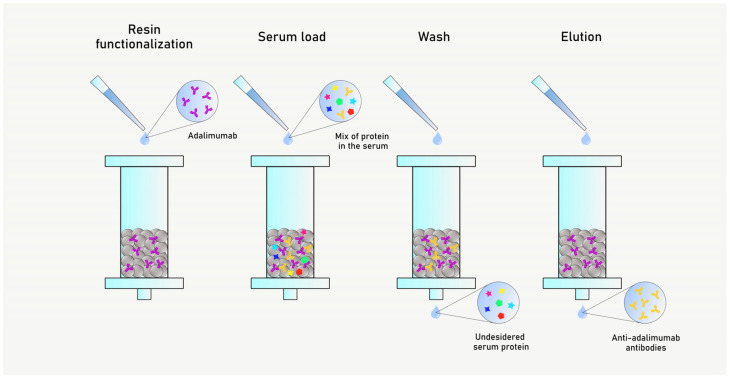
Scheme of anti-adalimumab antibodies (AAA) purification. Firstly, the resin is functionalized. Then, the serum is loaded, and a washing step allows to remove all the undesired and unbounded proteins. Lastly, the AAA are eluted.

AAA were purified from a patient’s serum showing high AAA concentrations. In order to obtain purified AAA fractions against either Humira^®^ and Hyrimoz^®^, two different CNBr-activated Sepharose resins were employed independently. Firstly, the resins were washed with HCl 1 mM to remove the lactose used for resin stabilization, centrifuged at 4000 rpm for 3 min, then washed with MilliQ water and coupling buffer (NaHCO_3_ 0.1 M, NaCl 0.5 M, pH 8.3). Humira^®^ or Hyrimoz^®^ were diluted in coupling buffer to a final concentration of 1 mg/mL and added to the resins overnight at room temperature. Adalimumab coupling reaction to the resin was monitored by UV absorbance at 280 nm before and after the resin functionalization, confirming the successful functionalization. The resins were washed with coupling buffer twice, and the non-reacted CNBr groups were blocked with a glycine solution 0.2 M pH 8.0 for two hours at room temperature. To remove the washing solution, the resins were washed with coupling buffer and acetate buffer (AcNa 0.1 M, NaCl 0.5 M, pH 4.3). Human serum samples were diluted 1:10 in D-PBS pH 7.2 and flowed through the columns for three times. Columns were washed with D-PBS pH 7.2 and coupling buffer to remove all the unbounded proteins. AAA were eluted with glycine 0.2 M pH 2.6 and the obtained fractions were neutralized with NaHCO_3_ 0.5 M (Figure 1).

Fractions were concentrated through Pierce™ Protein Concentrators PES, 30 K MWCO and the final AAA concentrations were calculated by evaluating their absorbance at 280 nm (Table 2) and using the Lambert–Beer law as follows:

A=ε×l×C

in which A = absorbance; ε = molar extinction coefficient (L × mol^−1^ × cm^−1^); l = optical pathway (cm); C = concentration (mol × L^−1^).

### 2.5. Kinetic Experiments

Purified AAA fractions at different initial calculated concentrations were diluted in HBS-EP+ running buffer to final concentrations of 16, 8, 4, 2, 1, 0.5, and 0.25 μg/mL. Kinetic experiments were conducted on the Biacore^®^ X100 using the multi-cycle kinetic analysis. Diluted samples were injected over immobilized adalimumab (Humira^®^, Hyrimoz^®^, and Imraldi^®^, respectively) for 120 s at a flow rate of 30 µL/min, following a dissociation phase of 600 s in which running buffer was injected for 600 s at a flow rate of 30 µL/min; finally, the chip surface was regenerated by injecting a 10 mM pH 2.5 glycine solution for 30 s and two injections of a solution of 15 mM NaOH and 1 M NaCl for 20 s, at a flow rate of 30 µL/min. A duplicate of the 0.25 µg/mL dilution was inserted at the end of each sample set to evaluate the sensor chip surface stability and reproducibility. Kinetic experiments were elaborated with Biacore^®^ X100 Evaluation Software version 2.0.2.

## 3. Results

With the idea in mind to purify anti-drug antibodies (AAA) from adalimumab-treated patients, and further study their kinetics among different biosimilars using the surface plasmon resonance technique, a screening of JIA patients was conducted.

### 3.1. Serum Samples Selection

Juvenile Idiopathic Arthritis patients were enrolled in the context of an ongoing study as part of regular medical care at the Pediatric Rheumatology Unit, AOU Meyer (Florence, Italy). All samples were screened to quantify ADL and AAA concentrations using the previously optimized SPR method [20]. Samples were selected for the present study if they met the following requirements: (1) presence of high level of AAA, (2) absence of free ADL drug, and (3) different ADL biologics used for the disease treatment. Two sera fulfilled these criteria, having no free drug, a high level of AAA, and being treated with two different adalimumab biosimilars (Hyrimoz^®^ and Imraldi^®^). Unfortunately, during the current study, no patients treated with Humira^®^ emerged to have AAA. The first patient was treated with Imraldi^®^, collected at month 6, with an AAA concentration over 40 μg/mL (Patient #1); the second one was treated with Hyrimoz^®^, collected at month 3, and presented an AAA titer over 40 μg/mL (Patient #2). One patient with no AAA was also selected for this study as control.

### 3.2. Anti-Adalimumab Antibodies Purification

**Figure 2:**
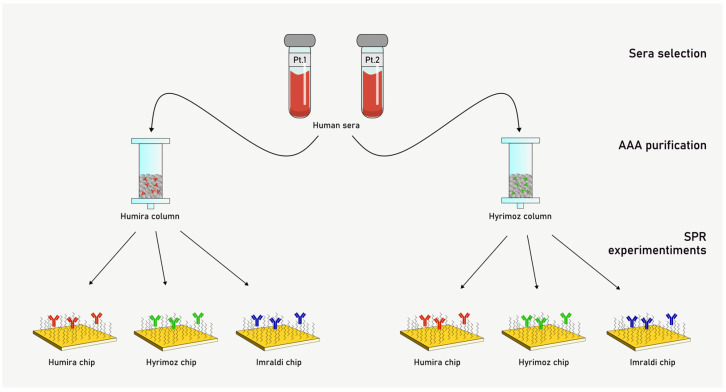
Scheme of the study with anti-adalimumab antibodies (AAA) purification and kinetic characterization.

To characterize AAA behavior among the different adalimumab formulations, we isolated AAA fractions from JIA patients’ sera using affinity columns functionalized with adalimumab. Unfortunately, the limited amount of serum available was enough for not more than two affinity columns purification; thus, we decided to select the originator, Humira^®^, and the biosimilar Hyrimoz^®^ as targets to be immobilized on the affinity columns. Then, two different Sepharose columns were functionalized with Humira^®^ and Hyrimoz^®^ separately, and the two serum samples were flowed over immobilized adalimumab columns. AAA fractions were collected and their concentration determined by UV spectrophotometry (Table 2). As a result, four different AAA purified fractions were obtained (Figure 2). As a negative control, the sample with no-detectable level of AAA was selected and flowed through both columns, and, as expected, no AAA were collected.

### 3.3. Surface Plasmon Resonance Experiments

The surface plasmon resonance sensor chips were prepared according to the standard amine coupling protocol. Humira^®^, Hyrimoz^®^, and Imraldi^®^ were immobilized independently on the active channel of each gold chip, using an irrelevant antibody on the reference channel to remove any undesired non-specific interaction. In order to obtain comparable results, the chips were prepared under similar conditions and the same target of immobilization. The three chips were considered to be equivalent and varied only in the adalimumab originator/biosimilar immobilized on the surface.

Anti-adalimumab antibody fractions, previously obtained by affinity chromatography, were flowed over each immobilized adalimumab originator/biosimilar in individual cycle of analysis based on the following steps: sample injection (association), washing with running buffer (dissociation), and subsequent chip surface regeneration.

Kinetic analyses were conducted following the multi-cycle kinetic approach, in which the samples were injected at increasing concentrations and the chip regeneration occurred between each sample injection. A replicate of a low-concentration sample was placed at the end of the sample set to assess the quality of the analysis. Obtained results were elaborated separately for each sample, fitting the experimental values to theoretical kinetic models.

**Figure 3:**
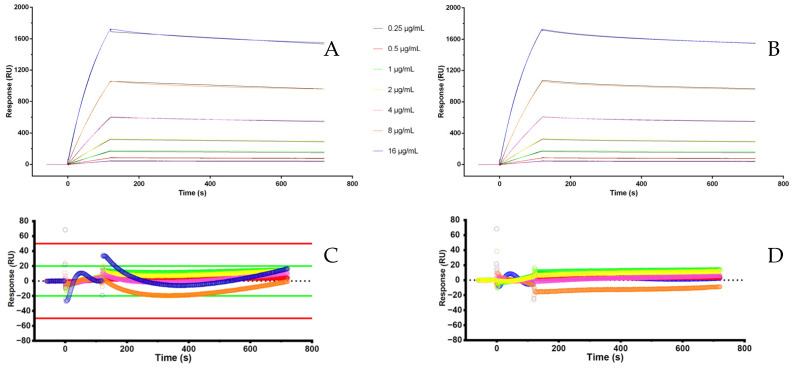
(**A**) Fitting with binding 1:1 model; (**B**) fitting with two state reaction model; (**C**) residuals for fitting A; (**D**) residuals for fitting B.

Individual analysis of each interaction yielded a high-quality fit with both the 1:1 binding kinetic model (Figure 3A) and the two-state reaction model (Figure 3B). According to the latter, following the initial interaction between AAA and adalimumab at the first binding site, engagement of the second site does not contribute to the SPR signal, as it involves only a conformational rearrangement of the bound analyte without a detectable change in refractive index or mass on the sensor surface. In fact, the “Check Data Components” software feature indicated a stronger interaction at the first binding site compared to the second. Then, the 1:1 binding model was further validated using three statistical criteria: (i) the residuals values between the experimental points and the theoretical ones, that are closely distributed along the zero (Figure 3C,D), (ii) low Chi^2^ values, and (iii) standard errors below 10% of the corresponding parameter values.

Therefore, the 1:1 binding model was employed to calculate the apparent affinity constant K_D_ (kd/ka), characterizing the interactions between each purified antibody fraction and each immobilized adalimumab formulation (Table 3). This model assumes a simple bimolecular interaction, in which a monovalent analyte binds to a monovalent ligand immobilized on the sensor surface, as exemplified by the following equation:

$$Ab+Lig\begin{array}{c} k_{a}\\ ⇋\\ k_{d} \end{array}Ab-Lig$$

### 3.4. Kinetic Summary

**Figure 4:**
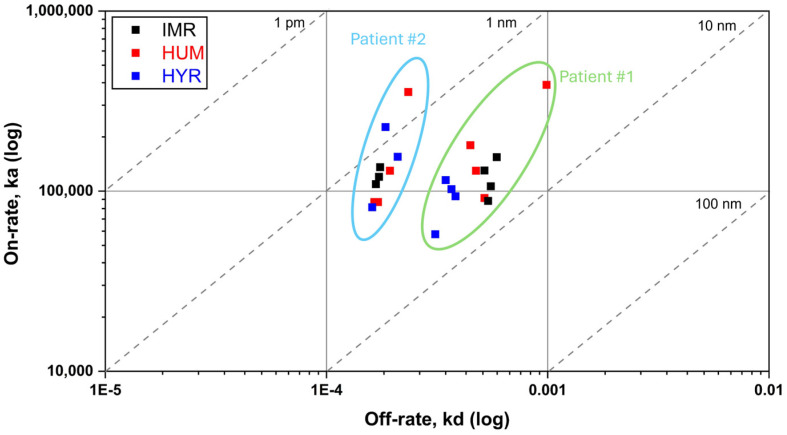
ka/kd graph for the 1:1 binding model summarizing the interactions between purified anti-adalimumab antibody fractions and the drugs Humira^®^ (red), Imraldi^®^ (black), and Hyrimoz^®^ (blue) in duplicate. K_D_ affinity constants (calculated as kd/ka ratio) are displayed on the diagonal lines.

The calculated kinetic parameters have been summarized in the ka/kd plot. This graph provides an overview of the kinetic properties of the interactions by plotting the association rate constant (ka) against the dissociation rate constant (kd) on logarithmic scales. Diagonal lines indicate apparent affinity constants (K_D_, calculated as kd/ka), then interactions with the same affinity, but different kinetics, are represented by points along the same diagonal line. In Figure 4 we reported the ka/kd plot for interactions between each purified AAA fraction and the adalimumab formulations. AAA interacted with Humira^®^, Hyrimoz^®^, and Imraldi^®^ with similar apparent affinity (10^−9^ M > K_D_ > 10^−10^ M); slight variations have been observed among the two patients, fewer among biosimilars (Figure 4).

## 4. Discussion

In clinical practice, the use of adalimumab biosimilars has become increasingly widespread, demonstrating their growing acceptance and efficacy as therapeutic alternatives to reference product Humira^®^. Overall, switching trials have consistently shown that transitioning from the reference Humira^®^ to a biosimilar does not significantly affect efficacy, safety, or immunogenicity, supporting the clinical interchangeability of biosimilars in treatment [6,21]. Adalimumab biosimilars have been demonstrated to be well tolerated with a safety profile similar to that of Humira^®^, judging the formulations to also have similar pharmacokinetics [22]. Usually, adalimumab immunogenicity among biosimilars is evaluated by assessing the anti-drug antibody presence in sera. Despite AAA evaluation still being controversial due to variation among the different techniques [23], the incidence of AAA was similar independently of adalimumab formulation and was not impacted by switching and double switching between originator and biosimilar treatment [24]. Moreover, other analysis focused on immunogenicity over long-term use showed no difference among adalimumab formulations in rheumatoid arthritis patients by AAA determination using a chemiluminescence assay [25,26].

In this context, with the idea in mind that anti-adalimumab antibodies among patients and biosimilars can be elicited with different kinetic characteristics, we performed an in-depth analysis for the interaction between purified AAA from JIA pediatric patients and the adalimumab formulations of Humira^®^, Imraldi^®^ and Hyrimoz^®^. We were able to purify, by affinity columns, AAA in patients treated with biosimilars (Hyrimoz^®^ and Imraldi^®^), not only to Hyrimoz^®^ but also to the originator. Moreover, purified AAA fractions exhibited analogous kinetic interactions with all the adalimumab formulations employed. The calculated kinetic constants showed that despite the k_on_ and k_off_ rates differing among biosimilars, the resulting K_D_ remains in the same range with values practically comparable independently of the fitting model applied. These results evidenced that despite purified AAA having been developed to a specific adalimumab formulation (biosimilars, in this study), they were able to recognize other adalimumab formulations including originator with a practically identical kinetic profile.

Nonetheless this study does not aim to address clinical correlations with AAA detection, the two patients presenting AAA analyzed in this study were both treatment responders, as it has been already previously reported [23]. Conversely, other authors evidence that AAA can be predictive for future relapsing symptoms up to three years [27]. Of note, according to our data, one of the patients was treated in concomitance with methotrexate, thus raising concern regarding the protective role of MTX in modulating the AAA production [28]. However, these results might be a start-up point to test this technique in a larger and prospective cohort to elucidate the AAA presence in a clinic point of view.

One of the limitations of this study was the inability to purify antibodies to other biosimilars like Imraldi^®^, as there was a limited quantity of patient sera available. While this limitation may reduce the generalizability of the findings, the fact that the chip containing Imraldi^®^ was able to recognize AAA with similar results allows us to hypothesize that the conclusions drawn about its immunogenic profile are likely to align closely with those of Hyrimoz^®^ and the originator Humira^®^. Another significant limitation of this study is the relatively small sample size, which is a consequence of the complex process required for antibody purification. As a result, the limited number of patient samples reduce the statistical significance that can potentially introduce biases in the interpretation of results. A larger sample size would enhance the reliability and representativeness of these findings, allowing more robust conclusions to be drawn. In any case, this study showed for the first time the kinetic performance of AAA and paves the way for a better understanding of the immunogenicity and clinical behavior among adalimumab formulations.

## 5. Conclusions

In this study, anti-adalimumab antibodies have been successfully purified from JIA patients’ sera using two different formulations of adalimumab. Kinetic studies showed that calculated K_D_ values for purified AAA from patients treated with Imraldi^®^ or Hyrimoz^®^ were found to be comparable across all biosimilars tested, including the originator Humira^®^. This suggests that AAA affinity in treated patients for the respective adalimumab formulations is similar, indicating no significant differences in the immunogenic profiles of these treatments. The similarity in K_D_ values across the biosimilars and the originator supports the notion that, at the level of AAA-adalimumab binding, the biosimilars can be considered clinically comparable to Humira^®^. This finding contributes to the growing body of evidence suggesting that these biosimilars can be used interchangeably with the reference product, with similar immunogenicity outcomes.

## Figures and Tables

**Table 1:** Patients’ clinical characteristics.

	Patient #1	Patient #2
Age	17 years	10 years
JIA subtype	RF-negative polyarticular JIA	Oligoarticular JIA
Disease duration	10 years	8 years
Previous exposure to ADL (duration)	Yes (56 months)	Yes (22 months)
Type of ADA	Biosimilar (Imraldi^®^)	Biosimilar (Hyrimoz^®^)
Disease remission	Yes	Yes
Concomitant MTX (dose)	No	Yes (oral MTX 7.5 mg/week, equivalent to 5 mg/m^2^/week)
Adverse events at the last available follow-up	No	No

**Table 2:** Calculated concentrations of AAA purified fractions.

Sample	Humira^®^ Column	Hyrimoz^®^ Column
Patient #1	37.9 ± 0.7 µg/mL	128 ± 1 µg/mL
Patient #2	32.9 ± 0.7 µg/mL	33.6 ± 0.7 µg/mL

**Table 3:** Calculated apparent affinity constants K_D_.

	Humira^®^ Column			Hyrimoz^®^ Column		
Patient #1	Humira^®^ chip	Hyrimoz^®^ chip	Imraldi^®^ chip	Humira^®^ chip	Hyrimoz^®^ chip	Imraldi^®^ chip
Binding 1:1	3.1 ± 0.6 (19%)	3.3 ± 0.2 (6.1%)	3.9 ± 0.1 (2.6%)	4.1 ± 1.5 (37%)	4.6 ± 0.4 (8.7%)	6.1 ± 0.7 (11%)
Two state reaction	2.6 ± 0.2 (7.7%)	1.59 ± 0.17 (11%)	2.4 ± 0.2 (8.3%)	2.6 ± 0.6 (23%)	2.6 ± 0.4 (15%)	4.0 ± 0.4 (10%)
Patient #2	Humira^®^ chip	Hyrimoz^®^ chip	Imraldi^®^ chip	Humira^®^ chip	Hyrimoz^®^ chip	Imraldi^®^ chip
Binding 1:1	1.13 ± 0.40 (35%)	1.10 ± 0.3 (25%)	1.41 ± 0.08 (5.7%)	2.0 ± 0.2 (10%)	2.7 ± 0.7 (26%)	1.73 ± 0.15 (8.7%)
Two state reaction	0.55 ± 0.07 (13%)	0.7 ± 0.2 (29%)	0.57 ± 0.14 (25%)	0.90 ± 0.40 (44%)	1.8 ± 0.4 (22%)	0.40 ± 0.05 (13%)

## Data Availability

The raw data supporting the conclusions of this article will be made available by the authors on request.

## Abbreviations

The following abbreviations are used in this manuscript:

ADL	adalimumab
AAA	Anti-adalimumab antibodies
SPR	Surface plasmon resonance
TNF-α	Tumor necrosis factor-alpha (TNF-α)
FDA	Food and Drug Administration
EMA	European Medicines Agency
JIA	Juvenile Idiopathic Arthritis
NHS	N-hydroxysuccinimide
EDC	1-ethyl-3-(3-dimethylaminopropyl)-carbodiimide
RU	Resonance Units
MTX	Methotrexate

## References

[1] Coghlan J., He H., Schwendeman A.S. (2021). Overview of Humira^®^ Biosimilars: Current European Landscape and Future Implications. J. Pharm. Sci..

[2] Gibbon J.B., Laber M., Bennett C.L. (2023). Humira: The First $20 Billion Drug. Am. J. Manag. Care.

[3] GaBI Journal Editor (2019). Patent Expiry Dates for Biologicals: 2018 Update. GaBI J..

[4] Biosimilar Medicines: Overview | European Medicines Agency (EMA). https://www.ema.europa.eu/en/human-regulatory-overview/biosimilar-medicines-overview.

[5] Kirchhoff C.F., Wang X.M., Conlon H.D., Anderson S., Ryan A.M., Bose A. (2017). Biosimilars: Key Regulatory Considerations and Similarity Assessment Tools. Biotechnol. Bioeng..

[6] Bellinvia S., Cummings J.R.F., Ardern-Jones M.R., Edwards C.J. (2019). Adalimumab Biosimilars in Europe: An Overview of the Clinical Evidence. BioDrugs.

[7] Biosimilars in the EU—Information Guide for Healthcare Professionals. https://www.ema.europa.eu/en/documents/leaflet/biosimilars-eu-information-guide-healthcare-professionals_en.pdf.

[8] Biosimilars Approved in Europe. https://www.gabionline.net/biosimilars/general/biosimilars-approved-in-europe.

[9] Abitbol V., Benkhalifa S., Habauzit C., Marotte H. (2023). Navigating Adalimumab Biosimilars: An Expert Opinion. J. Comp. Eff. Res..

[10] Scrivo R., Castellani C., Mancuso S., Sciarra G., Giardina F., Bevignani G., Ceccarelli F., Spinelli F.R., Alessandri C., Di Franco M. (2023). Effectiveness of Non-Medical Switch from Adalimumab Bio-Originator to SB5 Biosimilar and from ABP501 Adalimumab Biosimilar to SB5 Biosimilar in Patients with Chronic Inflammatory Arthropathies: A Monocentric Observational Study. Clin. Exp. Rheumatol..

[11] Gros B., Plevris N., Constantine-Cooke N., Lyons M., O’Hare C., Noble C., Arnott I.D., Jones G.-R., Lees C.W., Derikx L.A.A.P. (2023). Multiple Infliximab Biosimilar Switches Appear to Be Safe and Effective in a Real-World Inflammatory Bowel Disease Cohort. United Eur. Gastroenterol. J..

[12] Lázár-Molnár E., Delgado J.C. (2019). Implications of Monoclonal Antibody Therapeutics Use for Clinical Laboratory Testing. Clin. Chem..

[13] Van Schie K.A., Hart M.H., De Groot E.R., Kruithof S., Aarden L.A., Wolbink G.J., Rispens T. (2015). The Antibody Response against Human and Chimeric Anti-TNF Therapeutic Antibodies Primarily Targets the TNF Binding Region. Ann. Rheum. Dis..

[14] van Schouwenburg P.A., van de Stadt L.A., de Jong R.N., van Buren E.E.L., Kruithof S., de Groot E., Hart M., van Ham S.M., Rispens T., Aarden L. (2013). Adalimumab Elicits a Restricted Anti-Idiotypic Antibody Response in Autoimmune Patients Resulting in Functional Neutralisation. Ann. Rheum. Dis..

[15] Bartelds G.M. (2011). Development of Antidrug Antibodies Against Adalimumab and Association With Disease Activity and Treatment Failure During Long-Term Follow-Up. JAMA.

[16] Vincent F.B., Morand E.F., Murphy K., Mackay F., Mariette X., Marcelli C. (2013). Antidrug Antibodies (ADAb) to Tumour Necrosis Factor (TNF)-Specific Neutralising Agents in Chronic Inflammatory Diseases: A Real Issue, a Clinical Perspective. Ann. Rheum. Dis..

[17] Wadhwa M., Bird C., Atkinson E., Cludts I., Rigsby P. (2021). The First WHO International Standard for Adalimumab: Dual Role in Bioactivity and Therapeutic Drug Monitoring. Front. Immunol..

[18] Cludts I., Spinelli F.R., Morello F., Hockley J., Valesini G., Wadhwa M. (2017). Anti-Therapeutic Antibodies and Their Clinical Impact in Patients Treated with the TNF Antagonist Adalimumab. Cytokine.

[19] Real-Fernández F., Cimaz R., Rossi G., Simonini G., Giani T., Pagnini I., Papini A.M., Rovero P. (2015). Surface Plasmon Resonance-Based Methodology for Anti-Adalimumab Antibody Identification and Kinetic Characterization. Anal. Bioanal. Chem..

[20] Di Santo A., Accinno M., Errante F., Capone M., Vultaggio A., Simoncini E., Zipoli G., Cosmi L., Annunziato F., Rovero P. (2024). Quantitative Evaluation of Adalimumab and Anti-Adalimumab Antibodies in Sera Using a Surface Plasmon Resonance Biosensor. Clin. Biochem..

[21] Huizinga T.W.J., Torii Y., Muniz R. (2021). Adalimumab Biosimilars in the Treatment of Rheumatoid Arthritis: A Systematic Review of the Evidence for Biosimilarity. Rheumatol. Ther..

[22] Puri A., Niewiarowski A., Arai Y., Nomura H., Baird M., Dalrymple I., Warrington S., Boyce M. (2017). Pharmacokinetics, Safety, Tolerability and Immunogenicity of FKB327, a New Biosimilar Medicine of Adalimumab/Humira, in Healthy Subjects. Br. J. Clin. Pharmacol..

[23] Real-Fernández F., Pregnolato F., Cimaz R., Papini A.M., Borghi M.O., Meroni P.L., Rovero P. (2019). Detection of Anti-Adalimumab Antibodies in a RA Responsive Cohort of Patients Using Three Different Techniques. Anal. Biochem..

[24] Strand V., McCabe D., Bender S. (2024). Immunogenicity of Adalimumab Reference Product and Adalimumab-Adbm in Patients with Rheumatoid Arthritis, Crohn’s Disease and Chronic Plaque Psoriasis: A Pooled Analysis of the VOLTAIRE Trials. BMJ Open.

[25] Alten R., Markland C., Boyce M., Kawakami K., Muniz R., Genovese M.C. (2020). Immunogenicity of an Adalimumab Biosimilar, FKB327, and Its Reference Product in Patients with Rheumatoid Arthritis. Int. J. Rheum. Dis..

[26] Genovese M.C., Kellner H., Arai Y., Muniz R., Alten R. (2020). Long-Term Safety, Immunogenicity and Efficacy Comparing FKB327 with the Adalimumab Reference Product in Patients with Active Rheumatoid Arthritis: Data from Randomised Double-Blind and Open-Label Extension Studies. RMD Open.

[27] Huang B.-H., Hsu J.-L., Huang H.-Y., Huang J.-L., Yeh K.-W., Chen L.-C., Lee W.-I., Yao T.-C., Ou L.-S., Lin S.-J. (2025). Early Anti-Drug Antibodies Predict Adalimumab Response in Juvenile Idiopathic Arthritis. IJMS.

[28] Leinonen S.T., Aalto K., Kotaniemi K.M., Kivelä T.T. (2017). Anti-Adalimumab Antibodies in Juvenile Idiopathic Arthritis-Related Uveitis. Clin. Exp. Rheumatol..

